# Uncovering tau in wasteosomes (*corpora amylacea*) of Alzheimer’s disease patients

**DOI:** 10.3389/fnagi.2023.1110425

**Published:** 2023-03-30

**Authors:** Marta Riba, Jaume del Valle, Clara Romera, Raquel Alsina, Laura Molina-Porcel, Carme Pelegrí, Jordi Vilaplana

**Affiliations:** ^1^Secció de Fisiologia, Departament de Bioquímica i Fisiologia, Facultat de Farmàcia i Ciències de l’Alimentació, Universitat de Barcelona, Barcelona, Spain; ^2^Institut de Neurociències, Universitat de Barcelona, Barcelona, Spain; ^3^Centro de Investigación Biomédica en Red sobre Enfermedades Neurodegenerativas (CIBERNED), Madrid, Spain; ^4^Alzheimer’s Disease and Other Cognitive Disorders Unit, Neurology Service, Hospital Clinic, Institut d’Investigacions Biomèdiques August Pi i Sunyer, Universitat de Barcelona, Barcelona, Spain; ^5^Neurological Tissue Bank of the Biobanc-Hospital Clinic-IDIBAPS, Barcelona, Spain

**Keywords:** tau, wasteosome, Alzheimer’s disease, *corpora amylacea*, brain, biomarkers

## Abstract

Brain *corpora amylacea*, recently renamed as wasteosomes, are polyglucosan bodies that appear during aging and some neurodegenerative conditions. They collect waste substances and are part of a brain cleaning mechanism. For decades, studies on their composition have produced inconsistent results and the presence of tau protein in them has been controversial. In this work, we reanalyzed the presence of this protein in wasteosomes and we pointed out a methodological problem when immunolabeling. It is well known that to detect tau it is necessary to perform an antigen retrieval. However, in the case of wasteosomes, an excessive antigen retrieval with boiling dissolves their polyglucosan structure, releases the entrapped proteins and, thus, prevents their detection. After performing an adequate pre-treatment, with an intermediate time of boiling, we observed that some brain wasteosomes from patients with Alzheimer’s disease (AD) contained tau, while we did not detect tau protein in those from non-AD patients. These observations pointed the different composition of wasteosomes depending on the neuropathological condition and reinforce the role of wasteosomes as waste containers.

## Introduction

*Corpora amylacea* were first described in the human brain by Purkinje and Virchow in the 19th century (Virchow, 1854; Catola and Achúcarro, 1906). They have a high polysaccharide content (Sakai et al., 1969; Cavanagh, 1999) and can contain some proteins and other substances of neuronal, astrocytic and hematological origin as well as some related to infections (Martin et al., 1991; Singhrao et al., 1994; Schipper and Cissé, 1995; Selmaj et al., 2008; Meng et al., 2009; Libard et al., 2014; Pisa et al., 2016; Augé et al., 2018a; Riba et al., 2021a). Recently, it has been proposed that *corpora amylacea* participate in a mechanism involved in brain waste products removal (Riba et al., 2019, 2021a) and may be a manifestation of chronic glymphatic insufficiency (Riba et al., 2022b). Besides, in order to avoid the ambiguity of the term *amylacea* (which can indicate starch-like structures but also insoluble fibrillary proteins), it has been suggested to rename them as “wasteosomes,” emphasizing the waste products they entrap rather than their misleading amyloid properties (Riba et al., 2021a).

Although they were discovered almost 200 years ago, the study of their composition has been repeatedly and cyclically reporting inconsistent results (reviewed in Augé et al., 2018a). Some of these inconsistences are due to a huge methodological problem in the study of their composition by immunohistochemistry (IHC). In 2017 we observed that many commercial IgG antibodies used in IHC contain contaminant IgM antibodies due to the procedures used to obtain the antibodies. We also noticed that some of these IgM antibodies bind to wasteosomes. Thus, when the secondary antibody used in the IHC is not isotype-specific, false positive staining can arise due to the binding of the secondary antibody to the IgMs (Augé et al., 2017). A plausible explanation for the presence of these contaminant IgMs that bind to wasteosomes, and which are found in a very high number of commercial antibodies originating from different hosts, is that they are natural IgMs and, therefore, evolutionarily fixed and interspecific (Baumgarth et al., 2005; Ehrenstein and Notley, 2010; Holodick et al., 2017). Natural IgMs can bind to certain neoepitopes, which are epitopes that are formed *de novo* in tissues or structures that have to be removed, and which can be present in wasteosomes (Augé et al., 2019). These IgMs have been used to stain both wasteosomes and their equivalent structures in the mouse brain called periodic acid-Schiff (PAS) granules (Manich et al., 2015; Augé et al., 2017, 2018b,^2019^; Riba et al., 2019, 2021b,2022a; Wander et al., 2020, 2022). Recent findings have pointed out the carbohydrate nature of the neoepitopes of wasteosomes, although their specific structure remains unknown (Riba et al., 2021b).

In our abovementioned study of 2017, we studied the IHC staining of wasteosomes using two antibodies directed against the tau protein, specifically the Tau5 and the 5E2 antibodies. We used secondary isotype-specific anti-IgG antibodies that did not bind to IgMs. As the brain samples were from patients with Alzheimer’s disease (AD), the staining showed neurofibrillary tangles (NFTs) as well as positive neuronal axons, but no wasteosome staining was observed. The two antibodies also had contaminant IgMs that bound to wasteosomes. If we had used wide-ranging, non-isotype-specific secondary antibodies, we would have obtained a false positive staining of wasteosomes. We therefore concluded that wasteosomes did not contain tau protein, indicating the need to review previous studies (Augé et al., 2017).

However, a recent study by Wander et al. (2020), using IHC techniques and avoiding the false positive staining caused by IgMs, detected the presence of tau protein in wasteosomes. Interestingly, one of the antibodies used that gave a positive tau labeling in wasteosomes was the commercial Tau5 antibody, which did not appear to label wasteosomes in our previous study (Augé et al., 2017). In both our study and that of Wander et al. (2020), the vial of the Tau5 antibody was observed to contain IgMs that can give a false positive staining of wasteosomes. However, and after excluding IgM labeling, while staining with Tau5 antibody was not observed in wasteosomes in our work, it was observed in the study of Wander et al. (2020). The possible cause of this difference did not seem to be in the specific characteristics of the samples themselves, such as the variations in age, gender and the AD stage of the patient, as there was an overlap between the characteristics of the samples used in both studies. There were, however, some methodological differences between the studies, mainly in the protocols used for the IHC staining. Thus, for example, while Wander et al. (2020) performed antigen retrieval by boiling the samples at 100°C with citrate and incubating them on a hot plate, our group permeabilized the sections only with 0.1% Triton X-100.

In the present study, we delved even deeper into the immunolabeling of wasteosomes with the Tau1 and the Tau5 antibodies, reviewing, among other aspects, the protocols used by our group and Wander et al. (2020). The results on the presence or absence of tau labeling in wasteosomes will be addressed throughout the article. However, we anticipate that we detected and highlighted a new methodological problem in the study of wasteosomes. As with the IgMs, this methodological problem can also lead to errors in the study of wasteosomes and to misinterpretations of their function. Only by considering and readjusting these issues, the true nature and function of wasteosomes can be accurately understood.

## Materials and methods

### Human brain samples

Post-mortem human brain tissue was obtained from eight cases of neuropathologically confirmed AD and two cases of vascular encephalopathy (Table 1). Neuropathological examination was performed according to standardized protocols from the Neurological Tissue Bank (Biobank-Hospital Clínic-IDIBAPS, Barcelona) (Ximelis et al., 2021). Briefly, for each donor, a half brain was fixed in a 4% formaldehyde solution for 3 weeks, and 5 μm paraffin-embedded sections of at least 25 brain regions were used for the diagnosis. The other half brain was dissected in fresh. A hippocampal fragment was obtained and immersed for 24 h in 4% paraformaldehyde (4°C), followed by 48 h immersion in 30% sucrose in phosphate-buffered saline (PBS) (4°C). Afterward, once dried, the hippocampus fragment was frozen at −20°C and stored at −80°C. Finally, 6 μm sections were obtained in the cryostat to perform immunofluorescence (IF) or histochemical techniques. All these procedures were performed by the Neurological Tissue Bank (Biobank-Hospital Clínic-IDIBAPS, Barcelona), which provided the hippocampal samples.

**TABLE 1:** Medical data about the brain donors.

S	G	A	PMD	Neuropathological diagnosis^a^
1	F	80	24:30	AD (A3B3C3, Thal 5, Braak VI, CERAD frequent) + LBD
2	F	88	6:15	AD (A3B3C3, Braak V) + LBD
3	F	83	13:50	AD (A3B3C3, Braak VI, Thal 5, CERAD C)
4	M	86	7:15	AD (A2B1C1, Braak II, Thal 3, CERAD B) + ARTAG (frontobasal and temporomedial TSA) + AGD II + LBD (Braak 1, brainstem) + acute microinfarct
5	M	87	13:00	AD (A3B3C3, Braak V) + SVD
6	M	80	10:00	Vascular encephalopathy
7	M	70	04:30	Arteriosclerotic vascular encephalopathy
8	M	86	07:15	AD (A2B1C1, Thal 3, Braak II, CERAD B) + LBD + acute microinfarct
9	M	89	12:00	AD (A3B3C3, Thal 5, Braak VI, CERAD frequent) + severe CAA
10	M	91	04:30	AD (A3B3C3, Thal 5, Braak VI, CERAD frequent) + LBD

^a^AD: Alzheimer’s disease; AGD, argyrophilic grain disease; ARTAG, aging-related tau astrogliopathy; CAA, cerebral amyloid angiopathy; LBD, Lewy body dementia; SVD, small vessel disease; TSA, thorn-shaped astrocytes. S, subject; G, gender; F, female; M, male; A, age of death (years); PMD, post-mortem delay (in hh:mm).

All brain tissue samples were obtained from patients after they or their legal representatives gave written informed consent for the use of their brain tissue and their medical records for research purposes, as approved by the Ethics Committee of the Neurological Tissue Bank of the IDIBAPS Biobank, in accordance with the Declaration of Helsinki. All experiments involving human tissue were performed in accordance with appropriate guidelines and regulations and were approved by the Bioethical Committee of the Universitat de Barcelona (IRB00003099).

### Antigen retrieval

Hippocampal sections were air dried for 10 min at room temperature. They were then placed in a water bath set at 100°C inside staining dishes containing PBS (pH 7.2) or citrate buffer (pH 6.0), for 0, 5, 10, 20, 30 or 40 min. The staining dishes containing the samples were then taken from the water bath and left at room temperature for 20 min. After cooling down, the samples were washed with PBS.

### Immunofluorescence

Hippocampal sections were blocked and permeabilized with 1% bovine serum albumin (Sigma-Aldrich, Madrid, Spain) in PBS (blocking buffer, BB) containing 0.1% Triton X-100 (Sigma-Aldrich) for 20?min. Samples were then washed with PBS and incubated for 21 h at 4°C with the primary antibodies for simple or double staining. To detect tau, the primary antibodies used were mouse monoclonal IgG_1_ against tau (clone Tau5; 1: 200; AHB0042; Thermo Fisher Scientific, Rockford, IL, USA) and mouse monoclonal IgG_2*a*_ against Tau1 (clone PC1C6; 1: 500; MAB3420; Merck Millipore, Darmstadt, Germany), which detects hypo-phosphorylated tau. To detect wasteosomes, a mouse monoclonal IgG_2*a*_ against p62 (1: 250; ab56416; Abcam, Cambridge, UK) was used. Although p62 is an autophagy selective adaptor that is found and can be identified in most cells, labeling of wasteosomes with an anti-p62 shows a large and distinctive accumulation of p62 on their surface with a highly intense labeling and a characteristic ring shape, which enables wasteosomes to be specifically and unmistakably distinguished from other autophagic structures stained with anti-p62 (Augé et al., 2018a; Riba et al., 2021b,2022a). The sections were then washed and incubated for 1 h at room temperature with the corresponding secondary antibodies: AF488 goat anti-mouse IgG_1_ (1: 250; A-21121; Life Technologies, Eugene, OR, USA) and AF555 goat anti-mouse IgG_2*a*_ (1: 250; A-21137; Life Technologies). Nuclei were stained with the Hoechst stain (2 μg/mL; H-33258; Fluka, Madrid, Spain) and the samples were washed and coverslipped with Fluoromount (Electron Microscopy Sciences, Hatfield, PA, USA). Staining controls were performed by incubating with BB instead of the primary antibody before incubation with the secondary antibody.

### Periodic acid-Schiff staining

Frozen hippocampal sections were stained with the PAS method according to the standard procedure described previously (Augé et al., 2017). Briefly, sections were fixed for 10 min in Carnoy’s solution (60% ethanol, 30% chloroform and 10% glacial acetic acid). They were then pre-treated for 10 min with 0.25% periodic acid (19324–50, Electron Microscopy Sciences) in distilled water, followed by a washing step for 3 min with distilled water. Samples were immersed in Schiff’s reagent (26052–06, Electron Microscopy Sciences) for 10 min and washed for 5 min with distilled water. Nuclei were counterstained for 1 min with a haematoxylin solution, according to Mayer (3870, J. T. Baker, Center Valley, PA, USA). Then, the samples were washed, dehydrated with xylene, and coverslipped with the Eukitt mounting medium (03989, Merck Millipore).

### Image acquisition and processing

**FIGURE 1:**
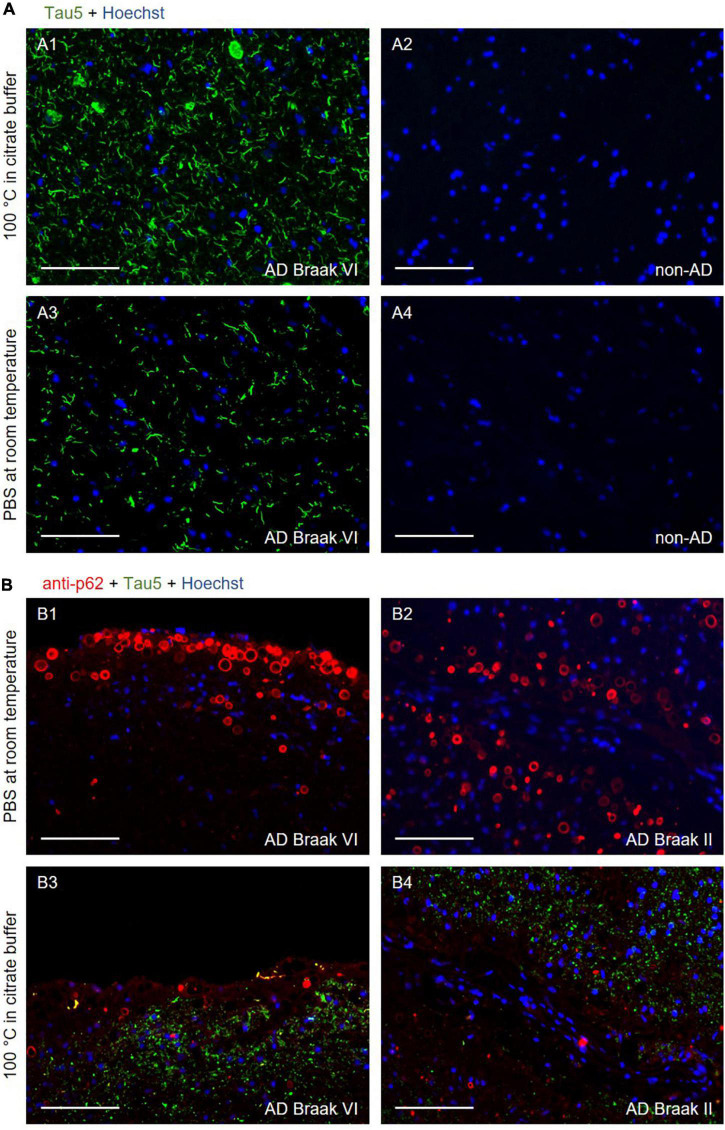
**(A)** Representative images of the CA1 region of the hippocampus from an Alzheimer’s disease (AD) (A1 and A3) and a non-AD (A2 and A4) patient stained with the Tau5 antibody (green). In A1 and A2, the samples were boiled for 40 min in citrate buffer before performing the immunostaining. In A3 and A4, no pre-treatment was performed, and the slides were maintained in PBS at room temperature. Tau staining was noted in the sections from the AD patient, with and without using antigen retrieval (A1 and A3, respectively). To enhance the visualization of tau staining in absence of antigen retrieval, the green channel of the A3 and A4 images was obtained with an exposure time that was three times longer than that of the A1 and A2 images. In any case, the absence of antigen retrieval could not be compensated by the extended time of exposure, as the pre-treatment increased not only the intensity of the tau staining, but also the number of structures stained. In the samples from the non-AD patient (A2 and A4), tau staining was not observed. A1–A4 were obtained from the same area. A1 and A3, and A2 and A4 come from consecutive sections. **(B)** Representative images of the hippocampus, near the fimbriodentate sulcus, from AD patients stained with the Tau5 (green) and anti-p62 (red) antibodies. In B1 and B2, where antigen retrieval was not applied, wasteosomes were stained with the anti-p62 antibody, whereas the tau staining was not discernible. In B3 and B4, where samples were boiled for 40 min in citrate buffer prior to the immunostaining, tau staining was observed, but wasteosomes were not observed with the p62 staining. B1–B4 were captured in the same area. B1 and B3, and B2 and B4 come from consecutive sections. In all **(B)** images, the green channel was obtained with an exposure time equal than that of the A1 image. Hoechst staining (blue) was used for nuclear staining. Scale bars: 100 μm.

**FIGURE 2:**
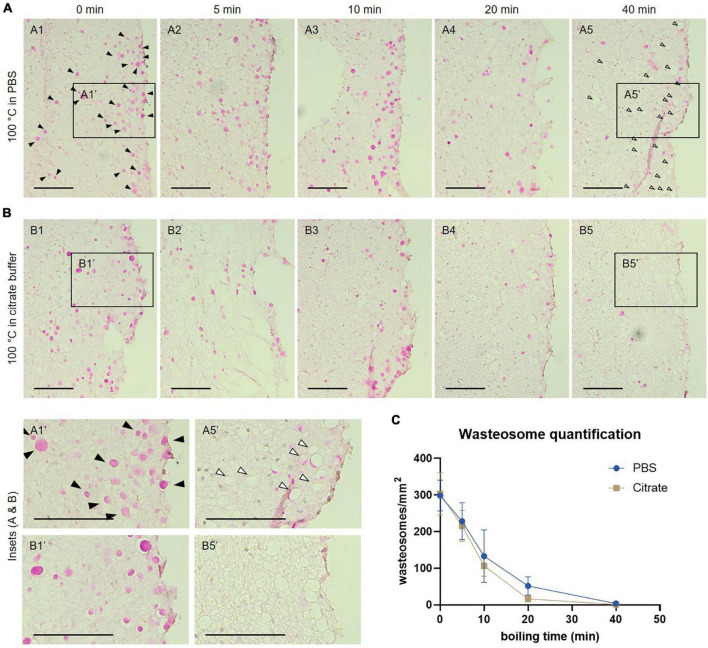
**(A)** Representative images of consecutive hippocampal sections (equivalent regions) from an Alzheimer’s disease (AD) patient boiled in PBS and stained with the periodic acid-Schiff (PAS) method. Samples were boiled in PBS for 0 (A1), 5 (A2), 10 (A3), 20 (A4) or 40 min (A5) before staining. **(B)** Representative images of consecutive hippocampal sections (equivalent regions) from an AD patient boiled in citrate buffer and stained with the PAS method. Samples were boiled in citrate buffer for 0 (B1), 5 (B2), 10 (B3), 20 (B4) or 40 min (B5) before staining. In A1, some wasteosomes stained with PAS are indicated by black arrowheads. Empty arrowheads in A5 indicate empty spaces compatible with dissolved or detached wasteosomes. A1′, A5′, B1′, and B5′ are the insets from A1, A5, B1 and B5, respectively. Scale bars: 100 μm. **(C)** Wasteosomes density at different boiling times with PBS or citrate buffer (mean ± SEM, *n* = 6 for each point). A decrease of the density of wasteosomes can be observed when boiling time increases. See text for details.

**FIGURE 3:**
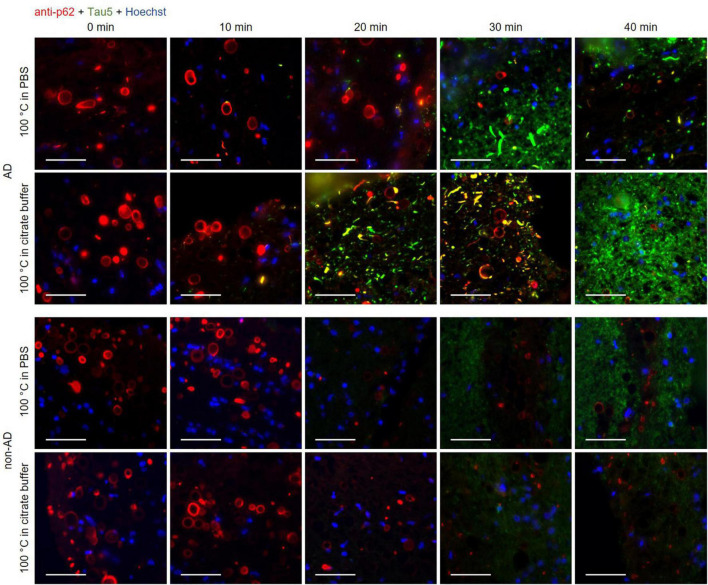
Representative images of hippocampal sections from an Alzheimer’s disease (AD) patient and a non-AD patient stained with the anti-p62 (red) and the Tau5 (green) antibodies. Each column corresponds to a different boiling time applied to the sample (0, 10, 20, 30 or 40 min). The first and second rows correspond to samples from an AD patient. The third and fourth rows correspond to samples from a non-AD patient. Samples in the first and third rows were boiled in PBS, while those in the second and fourth rows were boiled in citrate buffer. In control samples (0 min of boiling, first column), wasteosomes were stained with the anti-p62 antibody in the AD and the non-AD patients, but the tau staining was not discernible either in the wasteosomes or in the tissue. In the last column (40 min of boiling), the staining of the tau protein in the tissue of the AD samples was clearly discernible, but the p62 staining of the wasteosomes was absent. Only intermediate boiling times led to the observation of both p62 and tau staining, with some wasteosomes appearing to show the tau protein. The sections of each row were consecutive and the images shown were obtained from the same hippocampal region. Hoechst staining (blue) was used for nuclear staining. Scale bar: 50 μm.

**FIGURE 4:**
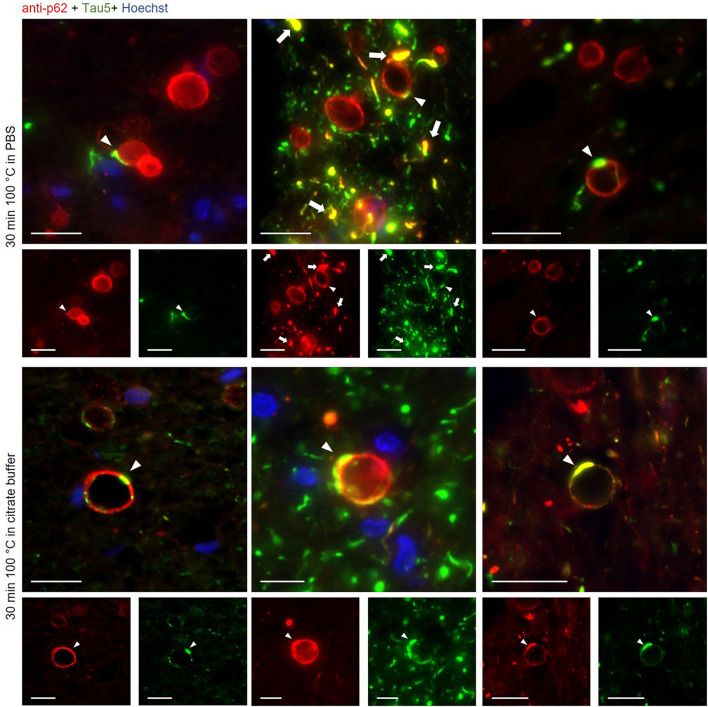
Representative images of wasteosomes from hippocampal sections of Alzheimer’s disease (AD) patients stained with the anti-p62 (red) and the Tau5 (green) antibodies after 30 min of boiling in PBS (first and second rows) or citrate buffer (third and fourth rows). Staining with the anti-p62 antibody showed the distinctive staining of wasteosomes. Tau staining colocalized with p62 staining in some areas of the wasteosomes (white arrowheads). p62 also colocalized with tau inclusions (white arrows). Hoechst staining (blue) was used for nuclear staining. Single channel (red and green) views are shown below merged images. Scale bar: 20 μm.

Images from Figures 1–4, 6 were taken with a fluorescence laser and optical microscope (BX41, Olympus, Hamburg, Germany) and stored in.tiff format. The images from IF were acquired using the same laser and software settings. The images in Figures 1, 2 were taken with the 20× objective and the images in Figures 3, 4, 6 were taken with the 40×. Exposure time was adapted to each staining and the respective control images were acquired with the same exposure time. Image treatment and analysis were performed with the ImageJ program (Schneider et al., 2012). Images that were modified for contrast and brightness to enhance their visualization were processed in the same way as the images corresponding to their respective controls. Images from Figure 1A correspond to the CA1 region, images from Figures 1B, 2, 3, 6 correspond to the fimbriodentate sulcus, and images from Figure 4 correspond to the fimbriodentate sulcus and hippocampal sulcus.

**FIGURE 5:**
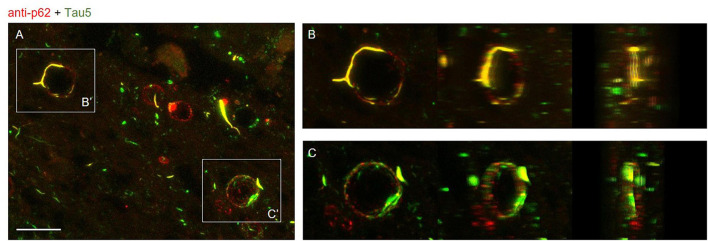
**(A)** Maximum intensity projection image obtained by confocal microscopy of an Alzheimer’s disease (AD) patient hippocampal section stained with the anti-p62 (red) and Tau5 (green) after 30 min of boiling the sample in citrate. Scale bar: 20 μm. **(B,C)** Frames obtained from Supplementary Videos 1, 2 where the colocalization of p62 and tau can be appreciated from different perspectives. These videos were obtained after a 3D reconstruction from insets B′ and C′ of the Z-stacks obtained by confocal microscopy.

In order to study the distribution of tau and p62 in wasteosomes, image stacks of stained slides were taken with a confocal laser scanning microscope at 63× (Leica TCS SP5, Leica Microsystems, Heidelberg, Germany) and 3D animations were obtained using the ImageJ program (Schneider et al., 2012; Figure 5 and Supplementary Videos 1, 2). Images correspond to the fimbriodentate sulcus.

To precisely indicate which hippocampal subfields from the whole section were sampled during imaging, we used the Hamamatsu NanoZoomer Digital Slide Scanner (40× magnification) to obtain low power microphotographs of a brain section from an AD case stained with the PAS technique (Supplementary Figure 1).

### Quantification and data analysis

After boiling consecutive hippocampal sections of 5 AD patients and a non-AD patient at 0, 5, 10, 20 or 40 min with PBS or citrate buffer and staining them with PAS, we counted the number of wasteosomes in a selected region of interest (ROI) of 0.13 ± 0.017 mm^2^ precisely located in the fimbriodentate sulcus (Figure 2 and Supplementary Figure 2) and calculated the density of wasteosomes per mm^2^ in each case and condition. Differences between groups were tested using a two-way ANOVA with Holm-Sidak’s multiple comparisons using GraphPad Prism software (GraphPad Software Inc., San Diego, CA, USA). *P* < 0.05 values were considered significant.

## Results

### Antigen retrieval enhances tau staining in brain tissue, but interferes with the staining of wasteosomes

As mentioned above, in a previous study (Augé et al., 2017), we were not able to detect tau staining in wasteosomes, contrary to the observations of Wander et al. (2020). Thus, we analyzed the divergences between our IF protocol and theirs to clarify the discrepancies. In the method performed by Wander et al. (2020), an antigen retrieval pre-treatment that involved incubating the samples in a citric acid-based solution was applied, boiling them for 10 min and incubating them on a hot plate for 10 min before performing the IF study. In our previous study, samples were only permeabilized with Triton X-100. In this regard, we wondered if the application of the pre-treatment affected the tau staining. Thus, we reanalyzed the possible presence of tau in wasteosomes using two different IF protocols: (1) our optimized IF procedure, and (2) our optimized IF procedure with an antigen retrieval step consisting of maintaining the samples in a water bath set at 100°C within staining dishes containing citrate buffer, pH 6.0, for 40 min.

As expected, the hippocampal sections from non-AD patients did not show positive tau immunostaining when using the Tau5 antibody with either of the two protocols (Figures 1A2, A4). On the other hand, as expected, the hippocampal sections from AD patients did show positive tau immunostaining when using the Tau5 antibody. In the case of the sections pre-treated with the antigen retrieval step, the characteristic tau staining was noticed in the NFTs and neuronal remains (Figure 1A1), while without antigen retrieval, tau staining was detected, although much weaker, in the large tau deposits (Figure 1A3). All these four images (Figures 1A1–A4) corresponded to the CA1 region of the hippocampus, which is an important area of tau accumulation in AD. It should be noted that the images corresponding to the sections not subjected to antigen retrieval were captured with an exposure time that was triple than the used for the samples subjected to antigen retrieval. Thus, as expected, antigen retrieval enhanced the staining of the tau protein present in the sections from AD patients. However, we did not observe, with either of the two protocols, any structure resembling or compatible with wasteosomes that were stained for tau, suggesting the absence of tau in the wasteosomes. However, the absence of staining in the wasteosomes made the precise observation of wasteosomes difficult.

Thus, we double-stained different sections from AD patients with the Tau5 and the anti-p62 antibodies. The staining with the anti-p62 antibody, which is a marker of wasteosomes (Augé et al., 2018a), would colocalize with the staining of tau if this protein is present in these structures. Figure 1B shows the double-stained sections from two different AD patients when antigen retrieval was not applied (Figures 1B1, B2) and when it was applied (Figures 1B3, B4). These images corresponded to the hippocampal region close to the fimbriodentate sulcus. In the absence of antigen retrieval, wasteosomes were clearly stained with the anti-p62 antibody, while the faint tau staining was not discernible in these images without an extended exposure time. Contrarily, after boiling for 40 min in citrate buffer, the staining of tau became evident in the parenchyma, but, unexpectedly, the staining of p62 in the wasteosomes did not appear. The information obtained from the images with and without antigen retrieval indicated that wasteosomes tended to accumulate mainly in the subpial regions, especially in higher numbers in the hippocampal sulcus and the fimbriodentate sulcus, while tau seemed to be present intraparenchymal, showing a high accumulation in CA1. However, what was intriguing was the absence of p62 staining after the antigen retrieval procedure.

One way to explain the disappearance of p62 is based on the fact that wasteosomes are polyglucosan bodies, formed mainly by aggregates of glucose polymers that solubilize upon boiling. Therefore, when applying the antigen retrieval step, the glucose polymer might dissolve and the proteins and other elements amassed in the polyglucosan scaffold might be discharged into the media. Accordingly, although the antigen retrieval procedure enhanced the staining of tau in the neuropil, we wondered if antigen retrieval interfered not only in the p62 staining of wasteosomes, but also in the staining of the possible tau present in wasteosomes. Consequently, the next step was to determine the effect of antigen retrieval on wasteosomes and, specifically, to study if boiling in citrate buffer or in PBS dissolved or solubilized the wasteosomes.

### Boiling pre-treatment causes wasteosome dissolving

Since wasteosomes were not stained with the anti-p62 antibody when applying the antigen retrieval procedure, we studied the possible effects of the boiling pre-treatment on wasteosomes. Specifically, we analyzed the polyglucosan skeleton of the wasteosomes by staining the hippocampal sections of 5 AD patients and a non-AD patient with the PAS technique, which detects the glycan fraction of wasteosomes, after boiling the samples for different periods of time in PBS. Specifically, we boiled consecutive sections for 0, 5, 10, 20 or 40 min, with those boiled for 0 min being the control or the procedure equivalent to no antigen retrieval. Additionally, we boiled equivalent samples in citrate buffer instead of PBS to compare the possible impact of each buffer (Figure 2 and Supplementary Figure 2).

At 0 min of boiling, the wasteosomes were clearly visible and stained with PAS in PBS (Figure 2A1) and in citrate buffer (Figure 2B1). In both series (Figures 2A, B), a decrease in the number of wasteosomes stained with PAS could be detected when the boiling time increased. For each patient and each experimental condition, the number of wasteosomes per mm^2^ was determined. The Figure 2C shows the density of wasteosomes per mm^2^ for each boiling time. A two-way ANOVA analysis revealed that the buffer used had no impact in the amount of wasteosomes stained (*p* > 0.05), but time had a significant effect (*p* < 0.05). Subsequent *post hoc* comparisons revealed that from 10 min onward, the amount of wasteosomes decreased in comparison with no boiling. The decrease of wasteosomes stained with PAS was accompanied by an increase of empty holes that were compatible with wasteosomes, as can be observed comparing the insets A1′ with A5′ and B1′ with B5′. These empty holes suggest the absence of any type of component in the regions previously filled by wasteosomes, and can explain why after a long time of boiling it is difficult to observe wasteosomes stained with anti-p62.

### Boiling time is relevant to stain tau and p62 in wasteosomes

The previous experiments indicate both the need of boiling the hippocampal sections in order to facilitate the tau staining and also that wasteosomes become altered or dissolved when performing the boiling pretreatment. Thus, we double-stained the hippocampal sections from AD and non-AD patients with the anti-p62 and the Tau5 antibodies after boiling the samples for different time periods (0, 10, 20, 30 and 40 min) with the intention of observing, at some intermediate times, both p62 and tau staining in the wasteosomes.

All these results are summarized in the images of Figure 3. As expected, the non-AD samples did not present tau staining at any of the tested boiling times (third and fourth rows). Moreover, and also as predicted in accordance with previous experiments, at 0 min of boiling we observed the presence of wasteosomes immunostained with the anti-p62 antibody. When increasing the time of boiling, the presence of wasteosomes stained for p62 decreased, and they were not observed at 40 min of boiling. With respect to the AD samples (first and second rows), at 0 min of boiling we observed the expected p62 staining in wasteosomes as well as the very weak tau staining located in the NFT and neuronal remains (Figure 3). On the other extreme, at 40 min of boiling, tau staining in the brain tissue was clearly visible, but the staining of p62 in wasteosomes was not observed. Interestingly, it was possible to observe, at the intermediate times of boiling (20 and 30 min), a small number of wasteosomes that stained with both the anti-p62 and Tau5 antibodies. Figure 4 shows representative wasteosomes from AD hippocampal sections with the combination of immunofluorescence stainings (anti-p62, Tau5 and Hoechst) at 30 min of boiling in PBS or citrate buffer. Moreover, the single channel images (green and red) are also supplied in order to better appreciate the colocalization between p62 and tau proteins. As can be seen, this colocalization is observed in the peripheral areas of the wasteosomes. Figure 5A shows the maximum intensity projection image obtained by confocal microscopy of an AD patient hippocampal section stained with the anti-p62 (red) and Tau5 (green) after 30 min of boiling the sample in citrate. The colocalization of p62 and tau can also be appreciated in the 3D reconstructions shown in the Supplementary Videos 1, 2 as well as in Figures 5B, C. It must be pointed that wasteosomes containing tau were observed in the sections coming from the AD patients 1, 9, and 10, but not in the sections obtained from the AD patient 8. Notably, regarding the histopathologic assessments of amyloid β deposits (A), staging of NFTs (B), and scoring of neuritic plaques (C) (ABC score; Montine et al., 2012), patients 1, 9, and 10 had an A3B3C3 score, while the score of the patient 8 was A2B1C1, indicating that patient 8 was in a lower Braak stage. Moreover, it is also remarkable that tau and p62 stainings colocalized not only in the periphery of these wasteosomes, but also in NFTs and neuropil threads present in the hippocampal tissue. All these points and their relevance will be commented in the discussion.

**FIGURE 6:**
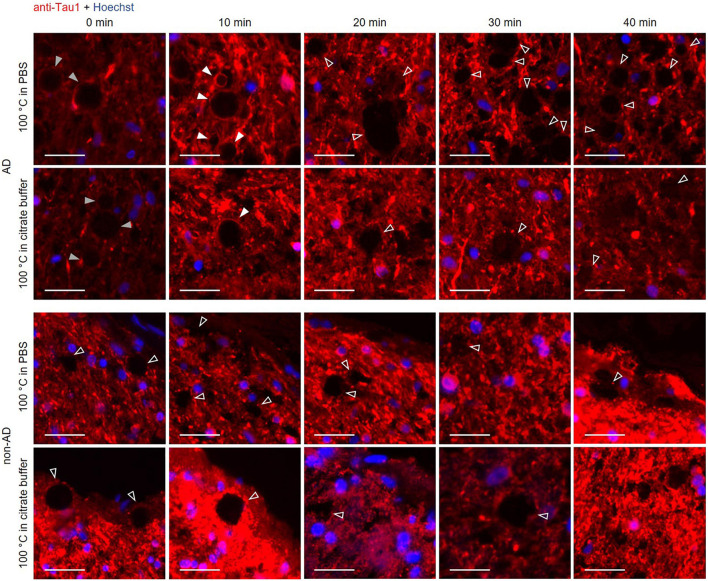
Representative images of hippocampal sections from an Alzheimer’s disease (AD) patient and a non-AD patient stained with the Tau1 antibody. Each column corresponds to a different boiling time applied to the sample (0, 10, 20, 30 or 40 min). The first and second rows correspond to samples from an AD patient. The third and fourth rows correspond to samples from a non-AD patient. Samples in the first and third rows were boiled in PBS, while those in the second and fourth rows were boiled in citrate buffer. At 0 min of boiling, the wasteosomes from the AD patient were slightly stained with Tau1 (gray arrowheads). The staining with Tau1 in wasteosomes from the AD patient increased at 10 min of boiling (white arrowheads) and disappeared from 20 min of boiling onward (empty arrowheads). Wasteosomes from the non-AD patient were not stained with Tau1 at any boiling time (third and fourth rows, empty arrowheads). The sections of each row were consecutive and the images shown here were obtained from the same hippocampal region. Hoechst staining (blue) was used for nuclear staining. Scale bar: 20 μm.

Since we observed a boiling time dependence in tau labeling with the Tau5 antibody, we tested another tau antibody to observe if the results followed the same dynamics (Figure 6). We used the anti-Tau1 antibody, which stains hypophosphorylated tau and, in the same way as with Tau5, we stained hippocampal sections from AD and non-AD patients after boiling the samples for 0, 10, 20, 30 or 40 min. As shown in Figure 6, at 0 min of boiling, the wasteosomes from the AD patient were slightly stained with anti-Tau1. The staining with anti-Tau1 in wasteosomes from the AD patient increased at 10 min of boiling and disappeared from 20 min of boiling onward. Wasteosomes from the non-AD patient were not stained with anti-Tau1 at any boiling time. Thus, Tau1 staining in wasteosomes can also be unmasked after a sensitive boiling time and disappears with prolonged boiling times, as with the staining with the Tau5 antibody. As expected, the parenchyma from both AD and non-AD patients showed Tau1 labeling under all conditions since the anti-Tau1 antibody has stringent specificity for the axons of neurons. In this case, no colocalization with p62 was performed because both primary antibodies were of the IgG_2a_ isotype obtained in mouse, not allowing a clear identification of this protein in wasteosomes.

## Discussion

Three different considerations arise from the present study: the first is related to several difficulties regarding the study of wasteosome composition, the second is linked to the presence of tau in wasteosomes from AD patients, and the third is related to the possible use of wasteosomes as a source of biomarkers for neurodegenerative diseases.

As indicated in the introduction, the study of the composition of wasteosomes by IHC procedures has generated inconsistent results. One important source of these discrepancies is the presence of contaminant IgMs in commercial antibodies and the false positive staining produced by them in wasteosomes (Augé et al., 2017). Here, we describe another methodological problem that shows up when using an antigen retrieval pre-treatment. The absence of antigen retrieval diminishes the detection of tau in the brain parenchyma and makes the detection of tau in wasteosomes even more troublesome. In this sense, both formic acid (Uemura et al., 2023) and boiling (Wander et al., 2020) have been used to unmask tau staining in wasteosomes. However, no comparison between these two methods has been performed yet. In this sense, the use of heat-induced antigen retrieval is a common procedure in IHC techniques (D’Amico et al., 2009), and our results report that an excessive boiling results in an effective tau staining of some structures like NFTs or neuropil threads, but also results in the progressive disappearance of tau staining in wasteosomes, as well as that of p62. As observed with the PAS staining, the glycan structure of wasteosomes dissolves with the boiling process and wasteosome staining ends up disappearing. Therefore, to detect tau in wasteosomes using boiling as antigen retrieval is necessary to use intermediate times in order to enhance tau staining but avoiding the complete dissolution of the wasteosomes. Indeed, some studies have already reported that a number of wasteosomes are not stained or disappear when the tissue sections are boiled (Sakai et al., 1969; Wander et al., 2020). Therefore, apart from the contaminant IgMs, another source of the inconsistences regarding the composition of wasteosomes is the antigen retrieval procedure based on boiling treatments, which is a common procedure in IHC staining and in which the boiling times differ between protocols and experimental settings.

In our previous studies we described that (1) the commercial Tau5 antibody contained IgMs that generated false positive staining in wasteosomes and (2) that tau was not present in wasteosomes (Augé et al., 2017). The first affirmation has been demonstrated as the IgMs present in the vials of the Tau5 antibody can actually generate false positive staining in wasteosomes if the secondary antibody is not isotype specific. In this sense, a wide distribution of tau was reported in all wasteosomes of both AD and non-AD patients (Singhrao et al., 1993) but in this study, dated in 1993, the authors used mouse anti-tau monoclonal antisera and a non-isotype specific anti-mouse IgG secondary antibody, which indicates that the staining is generated, at least in part, by the presence of IgMs. On the other hand, we must readjust the second affirmation, as we observed that tau can in fact be detected in some wasteosomes of AD patients, when using the adequate boiling time of antigen retrieval. In case of boiling the sample, the boiling time must be adequate and lower than the time required to dissolve the wasteosome polyglucosan structure. Moreover, it has to be pointed out that we did not find tau in wasteosomes from non-AD patients, which indicates that the composition of wasteosomes can be different according to the disease of the patient. In addition, we also observed that not all wasteosomes from AD patients contain tau. The common feature of all wasteosomes is their glycan structure, which becomes stained with the PAS technique and which corresponds to the scaffold of these containers (Riba et al., 2019, 2021b). In AD patients, in whom altered and insoluble tau aggregates in the brain, this protein is incorporated in the structure of some wasteosomes, mainly colocalizing with p62 protein, which is a waste adaptor commonly present in wasteosomes. Remarkably, the tau protein in NFTs and neuropil threads regularly colocalizes with the p62 protein. In any case, the tau protein is not a requirement for the formation of wasteosomes, since not all wasteosomes from AD patients contain tau and wasteosomes are also present in non-AD patients. Recent studies described the presence of tau protein in hippocampal wasteosomes from AD patients and control subjects (Wander et al., 2020). In our study, tau was only present in some wasteosomes from AD patients. The differences between the results of Wander et al. (2020) and those of the present work, apart from the previously mentioned antigen retrieval process, could be due to the processing of the sample after it was obtained. Specifically, Wander et al. (2020) used formalin-fixed paraffin-embedded samples, while we used cryopreserved samples. Briefly, paraffin embedding is known to better preserve morphological details, while cryopreservation is considered to better preserve antigenicity, post-translational modifications, such as phosphorylation, or enzymatic activity. All in all, after knowing that wasteosomes from AD patients may contain tau protein, further studies are needed to clarify the relationship between tau in wasteosomes and the ABC score, and especially the Braak stage. We hope in future studies to delve deeper into these aspects, which will certainly be relevant. On the other hand, we understand that it would also be interesting to determine the characteristics of the tau protein that accumulates in wasteosomes, since the anti-tau monoclonal antibody used (Tau5), although it is a monoclonal antibody, provides little information.

Tau is an extensively studied neuronal microtubule-associated protein that is comprised of a diverse range of fragments and isoforms in the central nervous system (Goedert et al., 1988). Tau is widely believed to stabilize microtubules in the axon (Dehmelt and Halpain, 2005; Kadavath et al., 2015), however, recent studies have revealed that tau allows axonal microtubules to have long labile domains, rather than acting as a microtubule stabilizer in the axon as tau dogma advocates (Qiang et al., 2018; Baas and Qiang, 2019). Besides, it is well-known that, in some neurodegenerative diseases, abnormal tau aggregates to form a range of pathological inclusions, such as NFTs, neuropil threads or dystrophic neurites that appear in AD (Kosik et al., 1986; DeTure and Dickson, 2019). These tau inclusions have been assumed to be associated with the redistribution of tau toward the neuronal soma in AD. Nevertheless, Paterno et al. (2022) have demonstrated that, in the brains of healthy adult controls, tau is more abundant in the cortical gray matter, which is enriched in neuronal soma and dendrites, rather than the cortical white matter, predominantly comprised of neuronal axons. Similarly, in AD brains, they have described that pathological tau aggregation is predominantly located in the cortical gray matter compared to the adjacent white matter as well. Taken together, these findings call into question the mechanisms of tau dissemination described in AD so far (Paterno et al., 2022).

In the development of AD, elevated hyperphosphorylated tau and its deposition are a better indication of disease progression and has a stronger relationship with a decline in cognition than Aβ42 or Aβ40 (Nelson et al., 2012; Aschenbrenner et al., 2018). However, although phosphorylated tau (p-tau) levels are high in the brains of AD patients, cerebrospinal fluid p-tau levels are weakly associated with the pathological changes indicative of NFTs in the brains of AD patients (Buerger et al., 2006; Seppälä et al., 2012).

Cerebrospinal fluid, owing to its direct connection with the extracellular space of the brain, is the most useful biological fluid reflecting the molecular events in the brain. Hence, research efforts have been made to develop biochemical biomarkers for the diagnosis of AD and other neurodegenerative diseases using cerebrospinal fluid. In recent studies, the cerebrospinal fluid p-tau/Aβ42 and Aβ42/Aβ40 ratios have been described to have high sensitivity, specificity, and overall diagnostic performance for intermediate-high AD neuropathologic changes (Mattsson-Carlgren et al., 2022). Furthermore, it has been observed that cerebrospinal fluid p-tau231 increases early in the development of AD pathology and has been proposed as a principal candidate for detecting incipient Aβ pathology for therapeutic trial applications (Ashton et al., 2022). Thus, although research efforts to obtain cerebrospinal fluid biomarkers for the diagnosis of AD seems to advance with time, there are other neurodegenerative diseases in which cerebrospinal fluid biomarkers are still not available. This is the case, for example, for the frontotemporal lobar degenerations (FTLD), that includes among others the FTLD-TDP, with the presence and aggregation of p-TDP-43 protein, and the FTLD-FUS with the fused in sarcoma (FUS) protein accumulation (Uemura et al., 2023). Unfortunately, some of these substances that could be useful for the diagnosis of certain neurodegenerative diseases are water insoluble and prone to aggregate, and are therefore hardly detectable in the cerebrospinal fluid.

An alternative to detect these insoluble substances originated in the brain under specific diseases is to analyze the composition of the wasteosomes, some of which are released from the brain to the cerebrospinal fluid and can be obtained from the pellet fraction when intraventricular cerebrospinal fluid samples are routinely centrifugated to eliminate cellular debris and dirtiness (Riba et al., 2019). In this sense, the observation shown in the present work, indicating the presence of tau in some wasteosomes of AD patients and their absence in the case of non-AD patients, reinforces the possible use of wasteosomes for diagnosis purposes. If wasteosomes could be obtained from cerebrospinal fluid collected by lumbar puncture, they could then be analyzed for the presence of certain components that can act as biomarkers. Actually, analyzing wasteosomes by more sensitive techniques such as protein mass spectrometry or matrix-assisted laser desorption/ionization-time of flight (MALDI-TOF) would allow more consistent and precise detection of proteins in wasteosomes and could shed light on the presence of some biomarkers in wasteosomes and their possible use as a diagnostic tool. Wasteosomes present an advantage over the supernatant of cerebrospinal fluid in that these structures, acting as waste containers, may include insoluble substances that precipitate and are therefore not detectable in the supernatant fraction.

In summary, in the present study, we highlight a new methodological problem in the study of wasteosomes, which occurs when pre-treatments of the tissue include boiling processes. Moreover, we observed that tau can be found in some wasteosomes from AD patients if samples are treated properly, but it is not present in wasteosomes from non-AD patients, thus pointing out that wasteosomes may differ from one another and that they may differ depending on the neuropathological condition. In addition, we reinforce the idea that wasteosomes, acting as waste containers that are expelled into the cerebrospinal fluid, may become a new diagnostic tool for neurodegenerative diseases.

## Data availability statement

The raw data supporting the conclusions of this article will be made available by the authors, without undue reservation.

## Ethics statement

The studies involving human participants were reviewed and approved by the Bioethical Committee of the Universitat de Barcelona (IRB00003099). The patients/participants provided their written informed consent to participate in this study.

## Author contributions

MR, JaV, CP, and JoV designed research, analyzed data, and wrote the manuscript. MR, JaV, CR, RA, LM-P, CP, and JoV performed research. All authors contributed to the article and approved the submitted version.
