# Supplementary figures and images for: Uncovering tau in wasteosomes (corpora amylacea) of Alzheimer’s disease patients
Source: Front Aging Neurosci. 2023 Mar 30;15:1110425. doi: 10.3389/fnagi.2023.1110425 (PMC10101234; doi:10.3389/fnagi.2023.1110425)

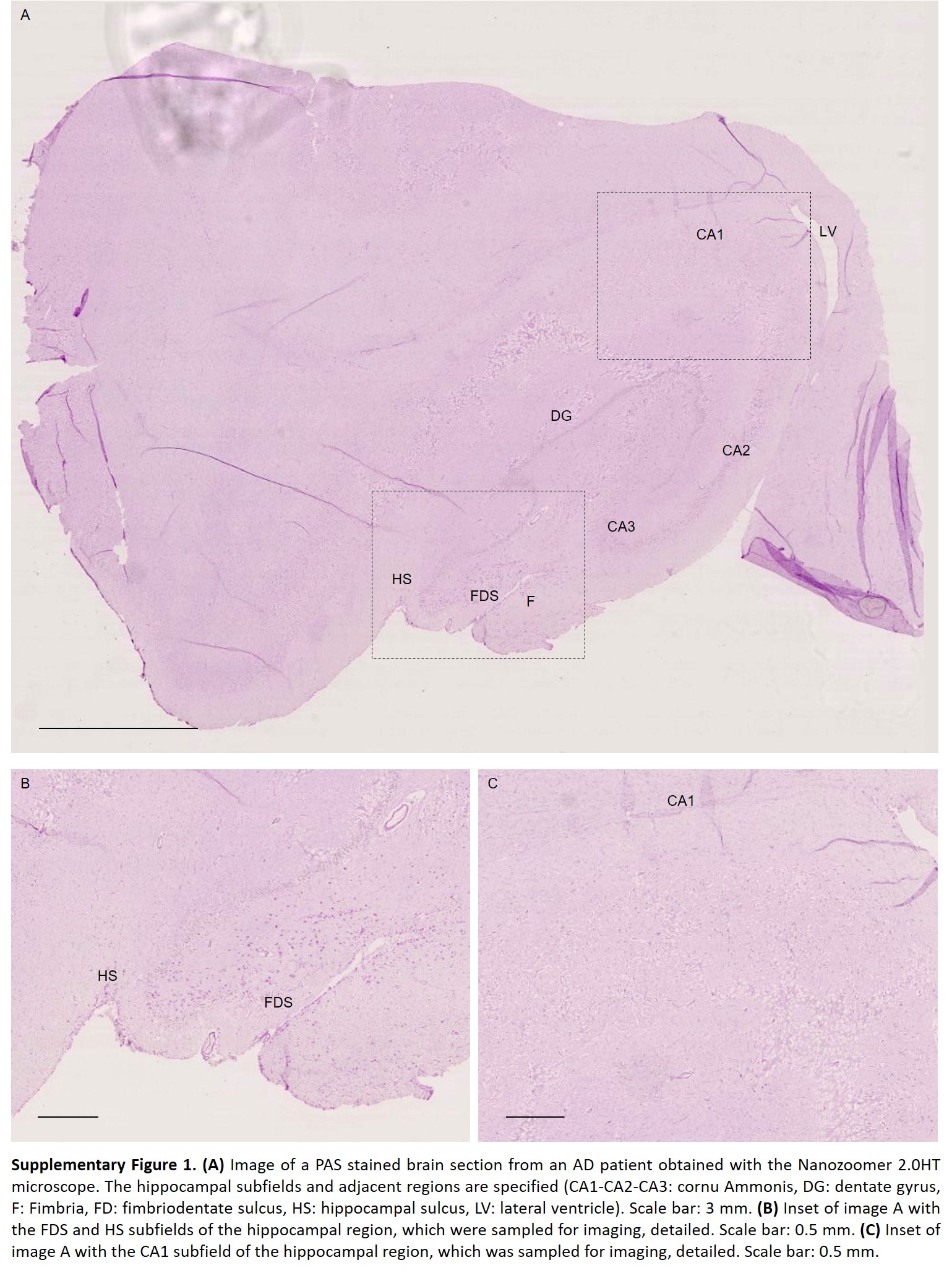

Supplement: Supplementary file 1 [file Image_1.JPEG]

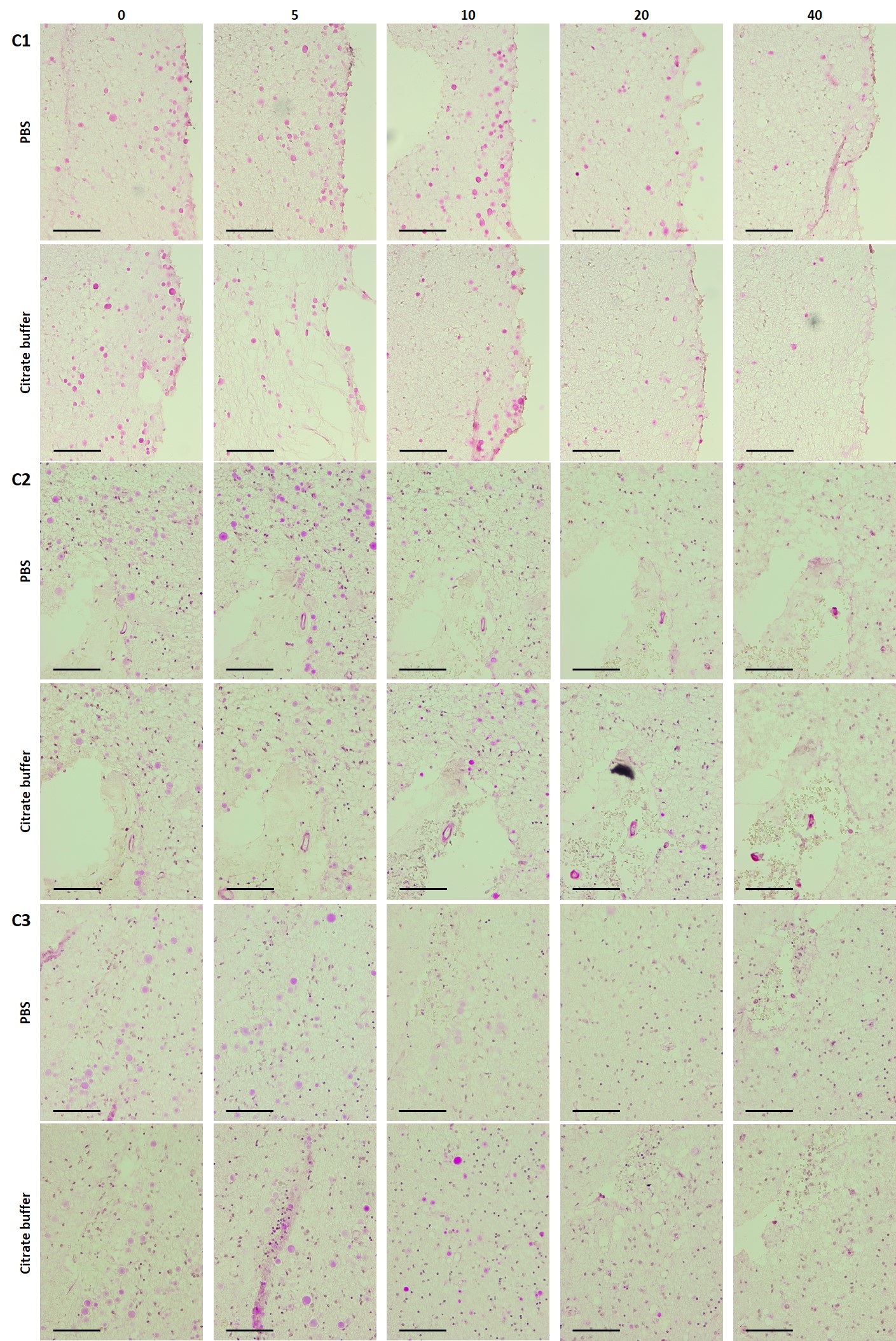

Supplement: Supplementary file 2 [file Image_2.JPEG]

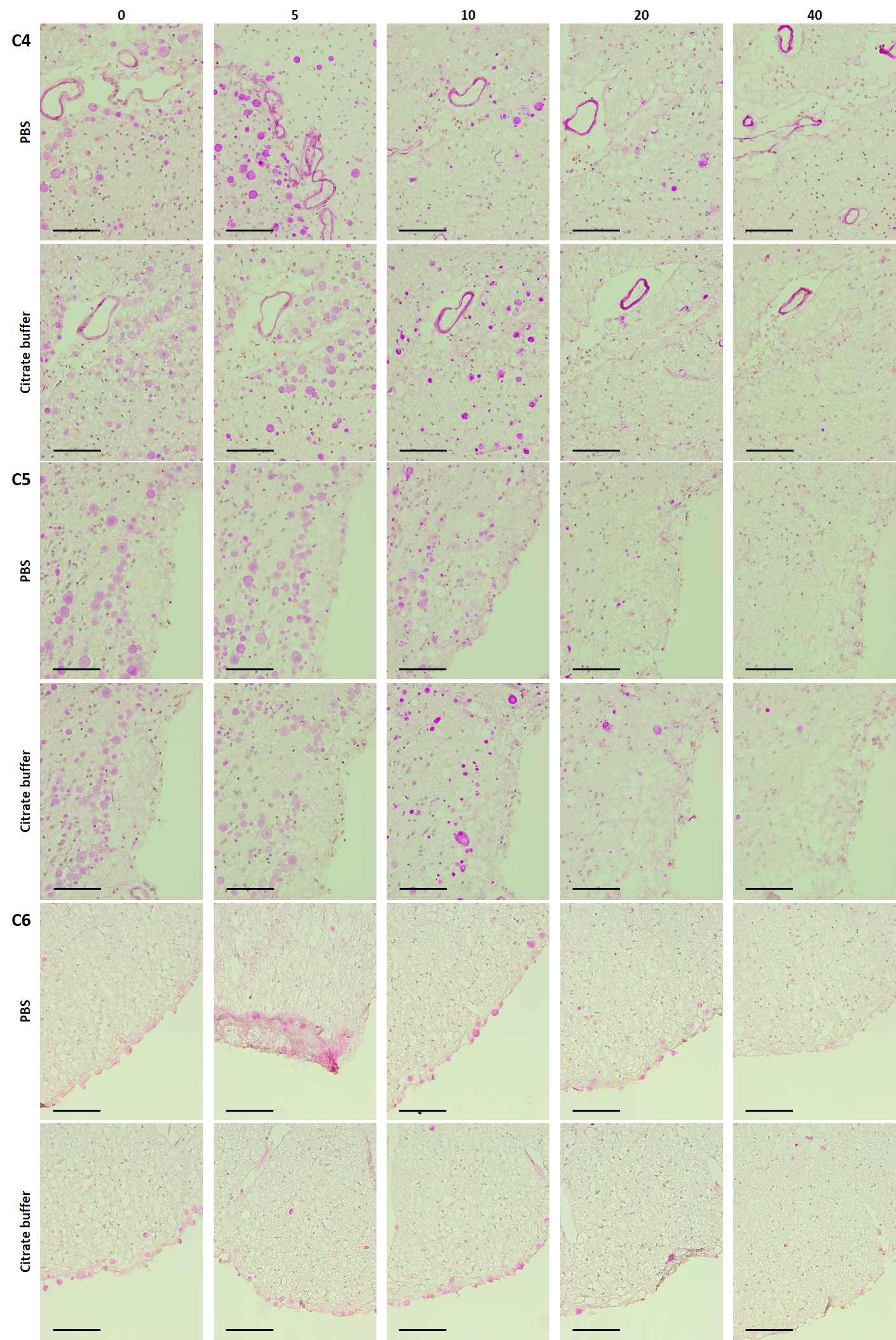

Supplement: Supplementary file 3 [file Image_3.JPEG]

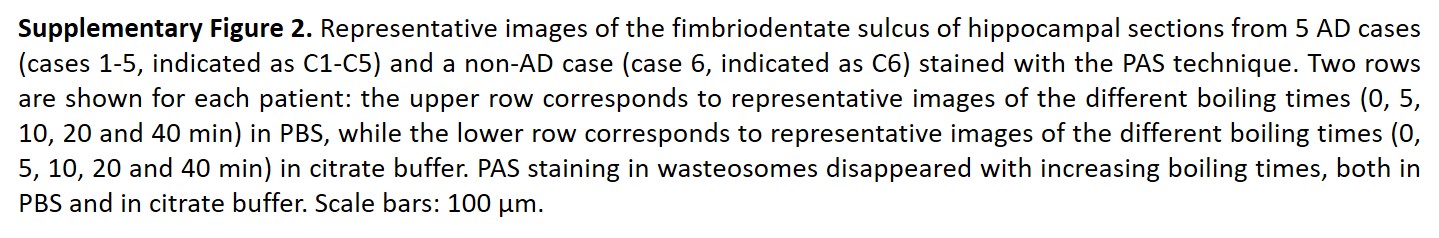

Supplement: Supplementary file 4 [file Image_4.JPEG]
